# Effect of Vasoactive Intestinal Peptide (VIP) on NKG2D Signal Pathway and Its Contribution to Immune Escape of MKN45 Cells

**DOI:** 10.1155/2013/429545

**Published:** 2013-10-20

**Authors:** Chong Wang, Xi-Jin Zhou, Yuan-yuan Li, Juan Wan, Le-ying Yang, Guo-Hua Li

**Affiliations:** Department of Gastroenterology, The First Affiliated Hospital of Nanchang University, Nanchang, Jiangxi 330006, China

## Abstract

*Objective*. To investigate VIP effect on the cytotoxicity of NK cell to gastric cancer cells *in vitro* and the relation between the effect with the NKG2D signal molecules in NK cells. *Material and Methods*. NK cells were purified from peripheral blood mononuclear cells (PBMC). Before and after NK cells were incubated with VIP or its antagonist (D-p-Cl-Phe6,Leu17)-VIP, we detected the cytotoxicity of NK cells to MKN45 gastric cancer cells by MTT and detected the expressions of NKG2D, DAP10, and NF-**κ**B proteins and mRNAs in NK cells by immunocytochemistry and RT-PCR in those conditions. Then we analyzed the effect of VIP and its antagonist on the cytotocicity of NK cell to gastric cancer cells and on expressions of NKG2D, DAP10, and NF-**κ**B signal molecules in NK cells. *Results*. VIP could inhibit the cytotoxicity of NK cells to MKN45 cells and could inhibit the expressions of NKG2D, DAP10, and NF-**κ**B in NK cells. However, (D-p-Cl-Phe6, Leu17)-VIP could reverse those effects. *Conclusions*. The VIP inhibited the cytotoxicity of NK cell to MKN45 cells which might get through inhibiting the expressions of NKG2D signal molecules in NK cells. This may be one mechanism of gastric cancer cells escaping organism immune clearance.

## 1. Introduction

Gastric cancer is the most common malignancy in gastrointestinal tract. The occurrence of gastric cancer must escape organism immune surveillance in order to be cleaned. The nonspecific immune cells take important role in direct cytotoxicity on tumor cells, especially the natural killer (NK) cells. The NK cells have a unique function, that is killing tumor cells or spontaneously transformed carcinoma cells without previous sensitization and MHC restriction [1]. So NK cells play a major role in the immune cleaning. Natural killer group 2 member D (NKG2D), which is the cell membrane activating receptor of some kinds of immune cells (especially NK cells), plays a key role in its cytotoxicity [2]. NKG2D has been shown to be important in the NK cell-mediated control of some cancers [3]. Human NK cells only express the long isoform of NKG2D, and it associates with DAP-10 to induce both a cytotoxicity and cytokine-mediated immune response [4, 5]. 

On the other hand, it has been known that gastric cancer cells could escape organism immunosurveillance by the following mechanisms [6]: (i) the expressions of tumor antigens were absent or decreased on the surface of tumor cells; (ii) the expressions of major histocompatibility complex (MHC) class I molecules on the tumor cells were reduced to a very low level; (iii) the tumor cells expressing FasL, which induced the apoptosis of lymphocytes that expressed Fas; (iv) the expressions of costimulatory signal molecules were absent in tumor cells; and (v) tumor cells could secrete some cytokines or hormones, such as VIP, IL-10, which inhibited organism immune function.

The gastrointestinal hormone vasoactive intestinal peptide (VIP) belongs to the secretin/VIP family, which was initially isolated from the intestine [7] and secreted by neurons, endocrine cells, immune cells [8, 9], and gastric carcinoma cells [10]. VIP affect not only gastrointestinal functions but also organism immune functions through binding to G protein-coupled receptors [11], which are distributed in most human tissues [12]. Many studies showed that VIP enhanced Th2 cells respond inhibited Th1 cell proliferation [13–15], impacted B cell differentiation [16, 17], and inhibited NK cell activity. So VIP is the most important immune inhibiting neuropeptide. Many studies showed that VIP was related with the gastric cancer. Hejna et al. reported that the serum VIP concentration increased in patients with gastric cancer [18]. We have showed that the gastric adenocarcinoma tissues contained secreting VIP cancer cells [10]. Above, this suggested that VIP might play an important role in inhibiting organism immune functions. 

So, we suppose that VIP, which is secreted by gastric cancer cells, may facilitate gastric cancer cells to escape organism immune cleaning by inhibiting NKG2D signal molecules in NK cells. To confirm it, we want to research the influence of VIP on NK cells cytotoxicity to kill the gastric adenocarcinoma cell line (MKN45 cells) *in vitro* and research the relationship of this influence with the NKG2D signal molecules in NK cells. 

## 2. Materials and Methods

### 2.1. Natural Killer (NK) Cells Separation, Purification, and Identification

Heparinized blood was collected between 6:00 and 10:00 am from 10 healthy volunteers of the staff at the Nanchang University (mean age, 42.6 ± 8.4; 60% male; no history of chronic illness and without current prescription medications usage). The peripheral blood mononuclear cells (PBMC) were obtained by standard Ficoll-Hypaque density gradient centrifugation and suspended by RPMI-1640 media with 10% heat-inactivated fetal bovine serum (FBS) (Gibco, Invitrogen, Grand Island, NY, USA). The activated NK cells were purified from PBMC by complement lysis (CDC) method [19] and cultured in RPMI-1640 medium with 10% newborn calf serum (NBCS) (Gibco, Invitrogen, Grand Island, NY, USA), 1 × 10^6^ U/L recombinant human interleukin-2 (rhIL-2, PeproTech, Rocky Hill, NJ, USA), and 10 mg/L phytohaemagglutinin (PHA, Sigma, St. Louis, MO, USA) under a microaerophilic environment (at 37°C, 2% O_2_, 5% CO_2_). The NK cells (expressed CD3^−^CD16^+^CD56^+^) percentage was detected by FACS after incubated with anti-human CD3 FITC/(CD16+CD56) PE Cocktail (BioLegend, San Diego, CA, USA) according to its instruction.

### 2.2. Cell Culture

The MKN45 cell line originated from human poorly differentiated gastric adenocarcinoma (from ATCC company). The cells were cultured in RPMI-1640 medium with 10% NBCS under a microaerophilic environment (at 37°C 2% O_2_, 5% CO_2_).

### 2.3. The Cytotoxicity of NK Cells on the Growth of MKN45 Cells

The MKN45 cells were cultured in the medium in a 96-well plate. Each well contained 200 *μ*L cancer cells solution in a 1 × 10^4^ cells/mL concentration. The supernate was thrown away after the MKN45 cells were cultured for 4 h, then each well was added NK cells and/or different drug solutions for different incubated time according to different groups as follows. Each plate included 4 wells for the negative controls (seeded by MKN45 and medium) and 4 wells for the blank controls (only seeded by 200 *μ*L medium). Each group included 4 wells at least in each time. Each test repeated three times. 

#### 2.3.1. Effect of VIP on the Growth of NK Cells to MKN45 Cells

In this test, the MKN45 cells in 96-well plate were added 180 *μ*L NK cells solution in a 1 × 10^5^ cells/mL concentration (the ratio of effect to target was 10 : 1) and 20 *μ*L VIP solutions to each well for 48 h. The final concentration of VIP in each well was 1 × 10^−5^ mol/L to 1 × 10^−7^ mol/L, respectively.

#### 2.3.2. Interaction between VIP and Its Antagonist

In this test, the MKN45 cells in 96-well plate were added, 180 *μ*L NK cells solution in a 1 × 10^5^ cells/mL concentration, and 20 *μ*L different drug solutions to each well for 48 h. The drug solution included VIP, its antagonist ([D-p-Cl-Phe6, Leu17]-VIP, Sigma, St. Louis, MO, USA), or VIP combined with its antagonist. The final concentration of VIP in each well was 1 × 10^−6^ mol/L, its antagonist in each well was 1 × 10^−4^, 1 × 10^−5^ or 1 × 10^−6^ mol/L, respectively. 

#### 2.3.3. MTT

The growth state of MKN45 cells in each test mentioned above were measured by methyl thiazolyl diphenyl tetrazolium bromide assay (MTT). The OD value (OD_490_) in MTT represented the quantity of living MKN45 cells, but not NK cells, because the suspending NK cells were thrown away in the course of removing supernate during MTT test.

### 2.4. Effect of VIP or Its Antagonist on NKG2D Signal Molecules of NK Cells

NK cells were cultured in the medium in a 6-well plate. Each well contained 2970 *μ*L NK cells solution in a 1 × 10^6^ cells/mL and 30 *μ*L different drug solutions to each well. The drug solution included VIP, its antagonist, or mixed solution of VIP and its antagonist. The final concentration of VIP and its antagonist in each well was 10^−6^ mol/L and 10^−5^ mol/L, respectively. After incubation for 48 h, the NK cells were used to detect target gene mRNA and protein expressions by RT-PCR or immunocytochemistry test. Each group included 3 wells at least in each test (negative control group contained 2970 *μ*L NK cells solution in a 1 × 10^6^ cells/mL concentration and 30 *μ*L medium). The test repeated three times.

#### 2.4.1. RNA Isolation and Reverse Transcription Polymerase Chain Reaction (RT-PCR)

*RNA Isolation*. After NK cells were incubated by the VIP and its antagonist for 48 h as mentioned above, the total RNA of the NK cells was extracted according to the procedure of the Trizol reagent (TianGen Biotechnology Co., Beijing, China).

*RT-PCR*. A typical 25-*μ*L reaction contained 10 *μ*L total RNA, 200 units of Moloney murine leukemia virus (MMLV) reverse transcriptase, 500 ng oligo(dT)_15_, 1 × 5 *μ*L reverse transcription buffer, 10 mmol/L deoxy-ribonucleoside triphosphate (dNTP), RNasin inhibitor (Promega, Madison, WI, USA), and ddH_2_O. The reaction was incubated for 1 h at 42°C and then heated at 95°C for 5 min to inactivate the reaction. The cDNA was used as a template for PCR amplification. The PCR for each cDNA was performed in a 25-*μ*L volume including 2 *μ*L template, 2 × Taq PCR MasterMix (TianGen Biotechnology Co., Beijing, China) 5 *μ*L, 0.3 *μ*mol/L each of target gene primers 1 and 2 and ddH_2_O under the following conditions: 95°C for 5 min, followed by 35 cycles at 94°C for 30 s, 55°C for 30 s (51°C for NKG2D and VPAC1, 52°C for VIP and NF-*κ*B, 54°C for DAP10), and 72°C for 1 min, finally 5 min at 72°C. The primers are listed in Table 1.

*Electrophoresis and mRNA Semiquantity*. The 4 *μ*L PCR product of each target mRNA was added with 1 *μ*L bromophenol blue buffer and was electrophoresed for 30 min on agarose gel stained with ethidium bromide (0.5 mg/mL). The length of the PCR products of VIP, VPAC1, NKG2D, DAP10, NF-*κ*B, and beta-actin was 361 bp, 432 bp, 365 bp, 282 bp, 326 bp, and 500 bp, respectively. The marker was DNAmarker DL600 (TianGen Biotechnology Co., Beijing, China). We examined and photographed each gel under UV light. The integrated optical density of each PCR product was measured by the HPIAS-1000 color picture analysis system (Olympus, Tokyo, Japan). The ratio of integrated optical density of the target gene to that of reference beta-actin was calculated, which represented the relative expression quantity of the target gene.

#### 2.4.2. Immunocytochemistry

After NK cells were incubated with the VIP, its antagonist for 48 h in 6-well plate as mentioned above, the NK cells were collected and were suspended to solution in 1 × 10^5^/mL concentration. The cell solution was dropped on slides and was fixed by 95% cold alcohol. After slides dried, the target proteins of NK cells were detected by immunocytochemistry test according to the procedure of the PV-9000 kit (for detecting VIP, DAP10, VPAC1, and NF-*κ*B) or PV-6003 kit (for detecting NKG2D) (Zhong Shan Company, Beijing, China). The primary antibodies of rabbit DAP10 polyclonal antibody, rabbit VPAC1 polyclonal antibody, mouse NF-*κ*B p65 monoclonal antibody, goat NKG2D polyclonal antibody (Santa Cruz Biotechnology, Santa Cruz, CA, USA), and rabbit VIP polyclonal antibody (Abcam, Cambridge, UK) were diluted to 1 : 100, 1 : 200, 1 : 50, 1 : 100, and 1 : 1000 with 0.01 mol/L PBS, respectively. The test set negative controls by replacing, respectively, the primary antibody with normal rabbit, mice or goat serum under the same experimental conditions. The positive particle was shown as dark brown under microscopy. The percentage of VIP, DAP10, VPAC1, NF-*κ*B, or NKG2D positive cells was the average percentage of positive cells in five randomly selected fields under a 200x magnification microscopy. 

### 2.5. Statistical Analysis

The statistical analysis was done by using SPSS 12.0 for windows (Chicago, IL, USA). The results were reported as mean ± SD. *t*-test was used to investigate the differences between two sessions. If ANOVA revealed a significant difference among three or more sessions, SNK-test was used to investigate the differences between any two sessions. Kruskal-Wallis *H*-test or Chi-square analysis was used for the assessment of enumeration data. 

## 3. Results

### 3.1. NK Cells Separation Efficiency (Table 2)

**Figure 1:**
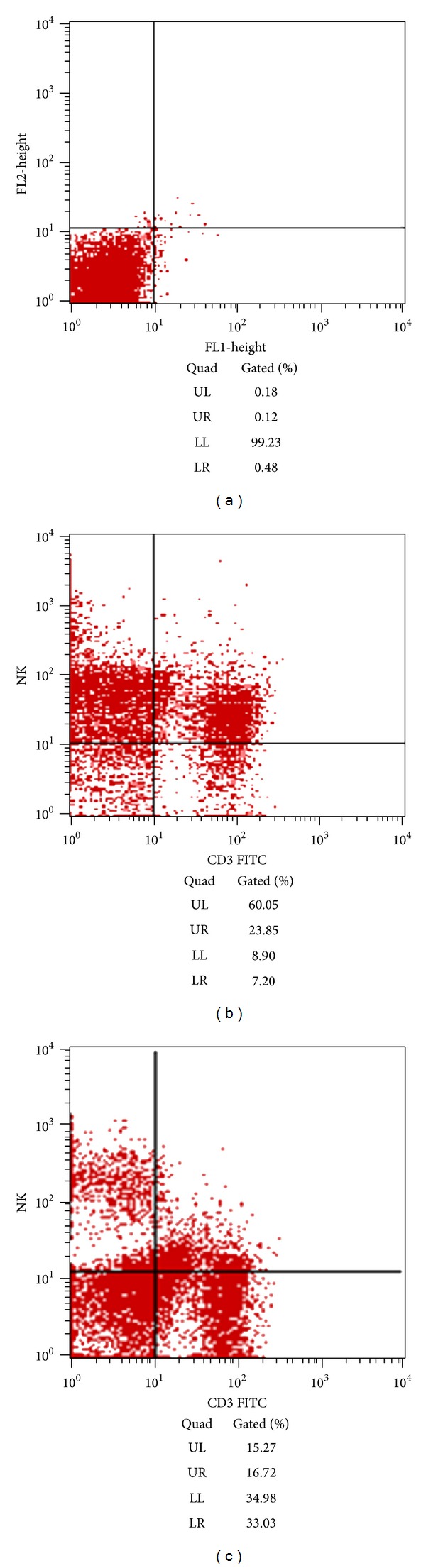
NK cells separation efficiency (a) negative control group, (b) NK cells purity by CDC method, and (c) NK cells purity in PBMC (UL represented CD3^−^/CD16^+^+CD56^+^ NK cells).

NK cells were purified from PBMC and identified by FACS. The purification of NK cells could reach 60% by CDC method (Figure 1).

### 3.2. The Cytotoxic Effect of NK Cells on the Growth of MKN45 Cells and VIP Effect on It (Table 3)

When MKN45 cells solution was incubated with different drugs and/or NK cells solution for 48 h, we found that OD_490_ value of MKN45 cells in only adding NK cells groups decreased more than that in adding NK cells with different VIP concentrations (*P* < 0.05). The higher VIP concentration was the more OD_490_ value was. 1 × 10^−6^ mol/L VIP could obviously inhibit the cytotoxicity of NK cells to MKN45 cells. However, there was no significant difference in the OD_490_ values between only adding different VIP groups with negative control group (*P* > 0.05).

### 3.3. Interaction between VIP and Its Antagonist (Table 4)

When MKN45 cells solution was incubated with NK cells solution and 10^−6^ mol/L VIP combined with different antagonist (1 × 10^−4^ mol/L, 1 × 10^−5^ mol/L, and 1 × 10^−6^ mol/L) for 48 h, we found that the OD_490_ values in the groups in presence of VIP and its antagonist were lower than that in the groups in present of VIP (*P* < 0.05). The more antagonist concentration was, the less the OD_490_ value was. That is to say, 1 × 10^−5^ mol/L antagonist could abolish the VIP influence completely. However there was no significant difference in the OD_490_ value between groups only added different antagonist with negative control group (*P* > 0.05).

### 3.4. VIP and Its Antagonist Influence on NK Cells' Cytotoxicity in the Different Time (Table 5)

When MKN45 cells solution was incubated with NK cells solution and/or the 1 × 10^−6^ mol/L VIP or 1 × 10^−5^ mol/L antagonist in wells for 24 h, 48 h, and 72 h, receptively, we found that there was no significant difference in OD_490_ values between only adding VIP or antagonist groups and control group from 24 h to 72 h (*P* > 0.05). There was significant difference in OD_490_ values between adding NK cells solution and control group from 48 h to 72 h (*P* < 0.05), but not 24 h (*P* > 0.05). The OD_490_ values in groups added VIP and NK cells were significantly higher than that in groups added only with NK cells in 48 h (*P* < 0.05), but not in 24 h or 72 h (*P* > 0.05). There was no significant difference in OD_490_ values between the groups added VIP, its antagonist and NK cells, and the groups only added NK cells from 24 h to 72 h (*P* > 0.05).

### 3.5. The Expressions of NKG2D, DAP10 and NF-*κ*B p65 mRNA and Protein

**Figure 2:**
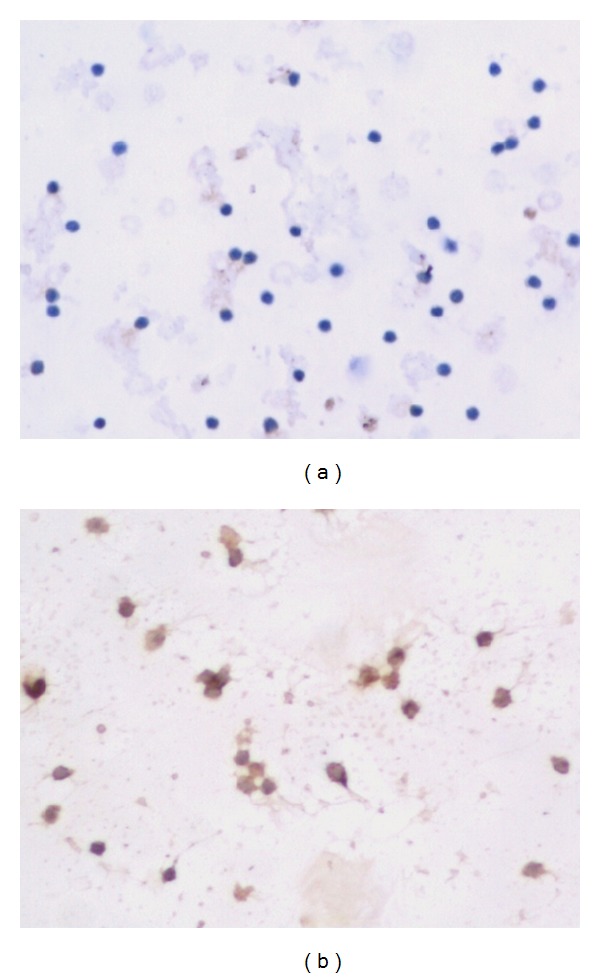
VIP and VPAC1 protein expression in NK cells ×200 ((a) showed VIP negative expression, and (b) showed VPAC1 positive expression).

**Figure 3:**
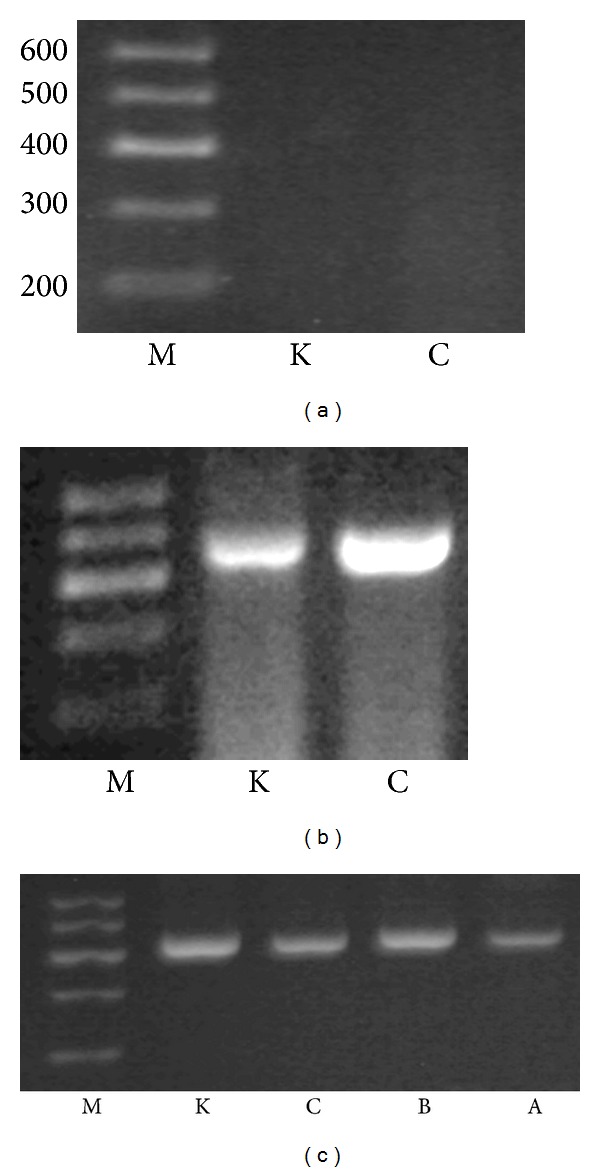
Electrophoresis of PCR product of VIP, VPAC1, and beta-actin mRNA in different groups. (a) stands for VIP, (b) for VPAC1, and (c) for beta-actin. M stands for marker DL600, K for MKN45 cells, C for NK cells, A for NK cells + VIP, and B for NK cells + VIP + VIP antagonist. The length of PCR products of VIP, VPAC1, and beta-actin was 361 bp, 432 bp, and 500 bp.

The expression of VIP mRNA and protein did not find in NK cells and MKN45 cells, however, VPAC1 could be detected in two kinds of cells (Figures 2 and 3). 

**Figure 4:**
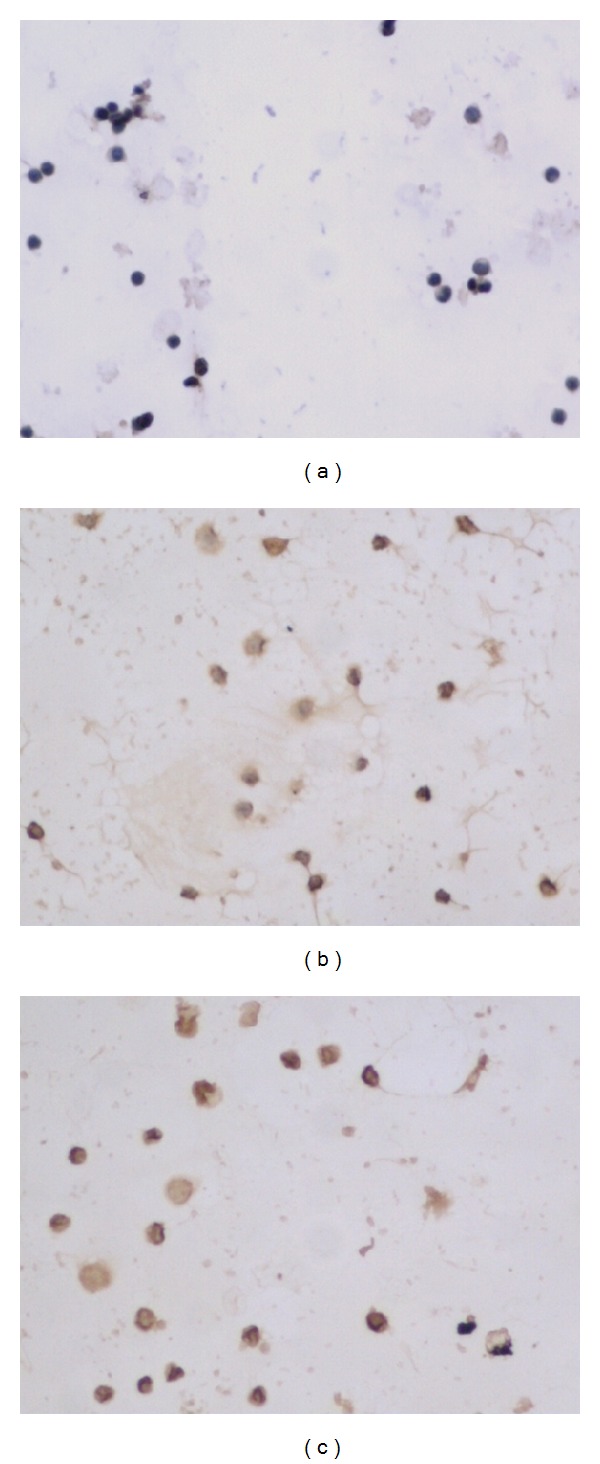
NKG2D protein expression in different groups ×200 ((a) NK + VIP, (b) NK + VIP + VIP antagonist, and (c) NK).

**Figure 5:**
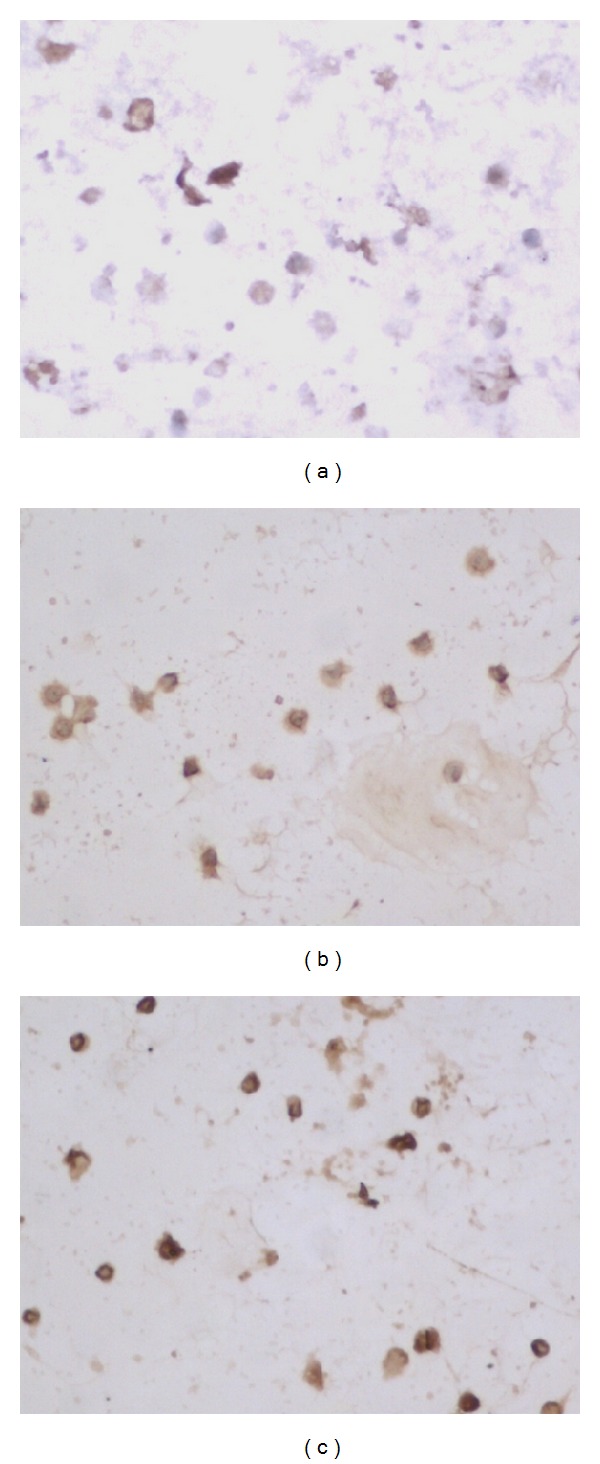
DAP10 protein expression in different groups ×200 ((a) NK + VIP, (b) NK + VIP + VIP antagonist, and (c) NK).

**Figure 6:**
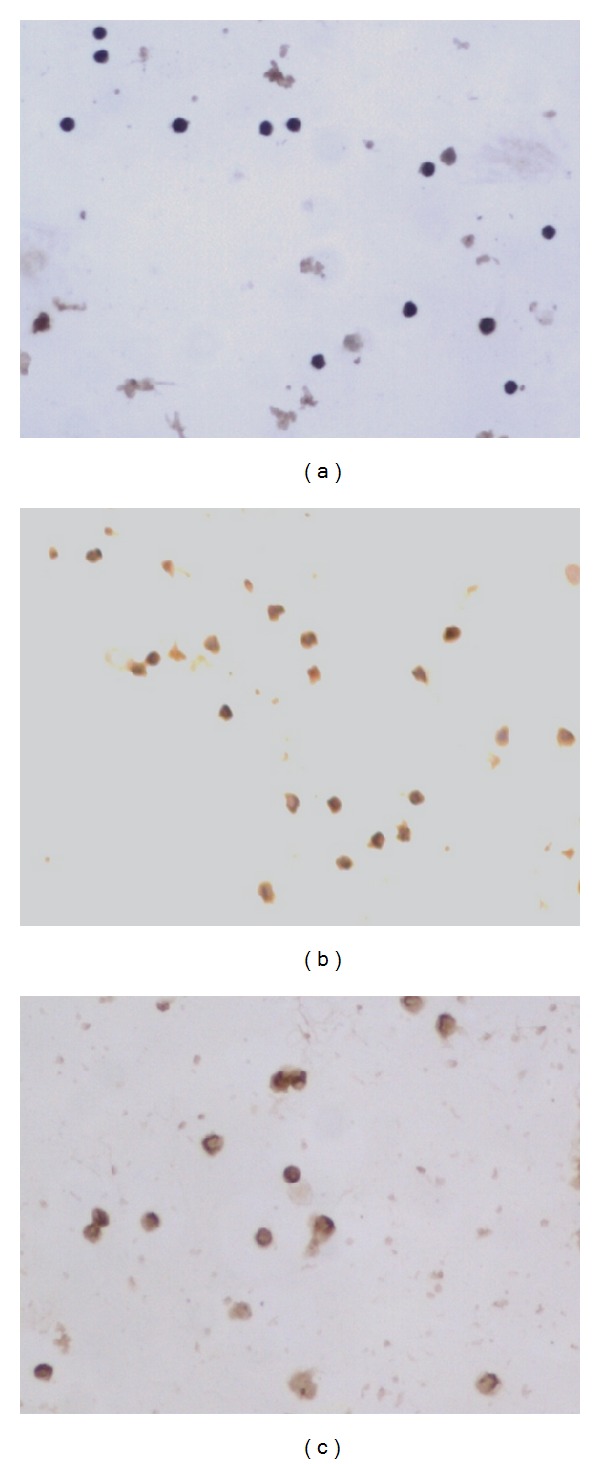
NF-*κ*B protein expression in different groups ×200 ((a) NK + VIP, (b) NK + VIP + VIP antagonist, and (c) NK).

**Figure 7:**
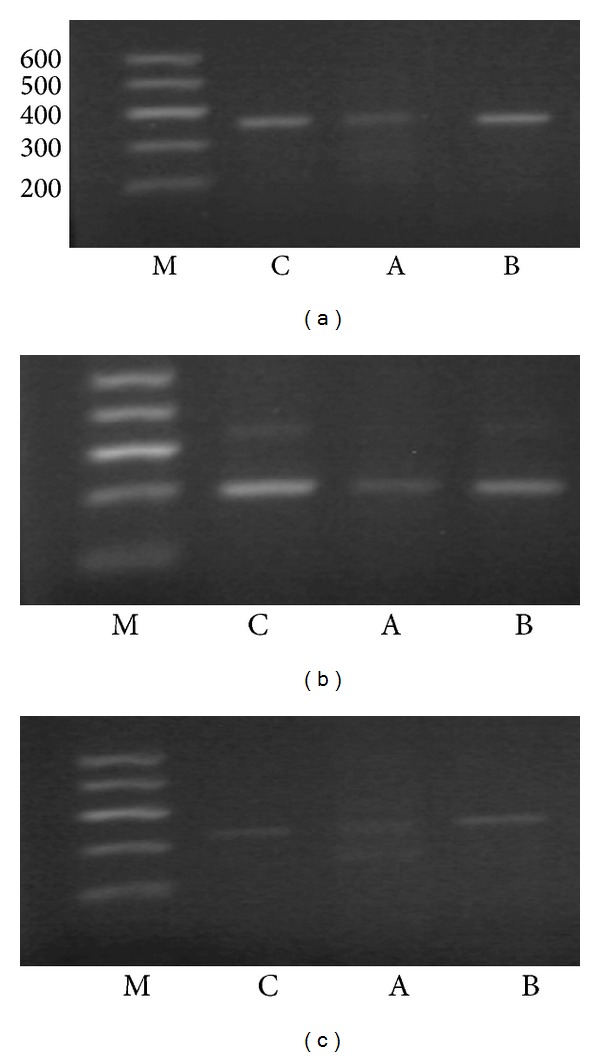
Electrophoresis of PCR product of NKG2D, DAP10, and NF-*κ*B mRNA in different groups. (a) stands for NKG2D, (b) for DAP10, and (c) for NF-*κ*B. M stands for marker DL600, C for NK cells, A for NK cells + VIP, and B for NK cells + VIP + VIP antagonist. The length of PCR products of NKG2D, DAP10, and NF-*κ*B was 365 bp, 282 bp, and 326 bp.

The expressions of NKG2D, DAP10 and NF-*κ*B mRNA and protein in NK cells decreased when NK cells incubated with VIP (*P* < 0.05, Table 6). VIP antagonist could partially or completely abolished the effect of VIP on the expressions of NKG2D, DAP10 and NF-*κ*B in NK cells (*P* < 0.05, Table 6, Figures 4, 5, 6 and 7).

## 4. Discussion 

Gastric cancer was once the second most common cancer in the world. In most undeveloped countries, the morbidity of stomach cancer had increased over the past half century. In China, stomach cancer was the most common malignant neoplasm. The gastric cancer cells must escape from organism immunosurveillance, which could clean the transformed tumor cells. In organism immunosurveillance system, the nonspecific immune cells, especially the NK cells, were the first line against tumor cells or virus infected cells and played an important role in directly cytotoxic effect on tumor cells. NK cells could kill tumor or spontaneous metastatic carcinoma cells without previous sensitization or MHC restricted [1]. In this study, we observed that NK cells had indeed cytotoxicity on tumor cells (MKN45, a kind of gastric adenocarcinoma cell line). However, how could gastric cancer cells escape from organism immune clearance? It was reported by the following mechanisms [6]: (i) the expressions of tumor antigens were absent or decreased on the surface of tumor cells; (ii) the expressions of major histocompatibility complex (MHC) class I molecules on the tumor cells were reduced to a very low level; (iii) the tumor cells expressed FasL, which induced the apoptosis of lymphocytes that expressed Fas; (iv) the expressions of costimulatory signal molecules were absent in tumor cells; and (v) tumor cells could produce cytokine or hormone, such as IL-10, which inhibited organism immune function.

Many studies showed that VIP was related with the gastric cancer [10, 18, 20]. We had showed that some gastric cancer cells in tumor tissues could secrete VIP [10]. The physiological functions of VIP in the GI tract were associated with the secretion and motor of the digestive system. Recently, many studies showed that VIP inhibited immune function. For example, VIP enhanced respondence of Th2 cells, inhibited Th1 cell proliferation [13–15], impacted B cell differentiation [16, 17], and inhibited NK cell activity through several pathways. So we wonder that if VIP, which was secreted by gastric cancer cells, facilitate gastric cancer cells to escape immunosurveillance by inhibiting NK cells activity. To confirm it, we designed the study. In our study, we observed that VIP could significantly inhibit the cytotoxicity of NK cells on MKN45 cells at the 10 : 1 ratio of effect to target and could be reversed completely by VIP antagonist. It confirmed our speculation.

It has been reported that endogenous VIP affect the growth of some gastric cancer cells [10] by binding to G-protein-coupled receptors [11, 21]. We observed that exogenous VIP and VIP antagonist did not affect the proliferation of MKN45 cells. So, we excluded the influence of VIP or antagonist on MKN45 cell. That is to say, VIP and antagonist affected OD_490_ of MKN45 by acting on NK cells. 

It has been know that the NK cells cytotoxicity was through the activating receptor of NK cells, NKG2D. NKG2D had been shown to be important in the NK cell-mediated control of some cancers. NKG2D was a C-type lectin-like type receptor and belonged to the NK group 2 (NKG2) of receptors as member D. Although NKG2D belonged to the NKG2 family, it did not share most of their properties. In contrast to other members of the family, NKG2D was a homodimer and recognized a number of stressed cells induced by MHC class I-like ligands [22, 23]. NKG2D as a molecular sensor could detect “induced self” on cells in danger, which was mostly triggered by viral infections and by some factors to cause DNA damage and tumor transformation [2]. NKG2D signal transduction should through two adaptor proteins, DAP10 and DAP12 [5], which were associated with the receptor as homodimers. DAP12 carried an immunoreceptor tyrosine-based activation motif (ITAM) [24]. DAP10 had a YINM motif in its cytoplasmatic tail [4, 5]. Human NK cells only expressed the long isoform of NKG2D (NKG2D-L), and it was associated with DAP-10 to induce immune response. It was reported that NKG2D signal pathway might touch upon other signaling molecules, such as nuclear factor-kappa B (NF-*κ*B) [25, 26]. 

In our study, we found that VIP downregulated the NKG2D, DAP10, and NF-*κ*B expression in NK cells, which could be reversed completely or mostly by VIP antagonist. Moreover, we found that the alteration trend of the expressions of NKG2D, DAP10, and NF-*κ*B p65 was similar, so NKG2D/DAP10/NF-*κ*B might be a pathway by which VIP affected NK cells. NF-*κ*B was a transcription factor, which was heterodimers or homodimers consisted of the members of the NF-*κ*B family. It was a critical regulator of multiple biological functions, such as affecting innate and adaptive immunity, affecting cell survival. So NF-*κ*B/NKG2D/DAP10 might be another pathway by which VIP affected NK cells. They need further confirmation.

In our study, we need to attain highly purified NK cells. It has been reported that NK cells could be separated by immunomagnetic beads sorting system [27] and flow cytometer (FCM) [28]. However, those methods mentioned above were expensive and complicated. In our study, we used complement lysis (CDC) method to separate NK cells. We found that the NK cells purity raised from 18% (before purification) to 60% (purified). This method was reported by Ciccone E [19]. He found that the purity, surviva rate, and cytotoxic activity of NK cells were good when using CDC method. There were T/B lymphocytes in the NK solution except NK cells, but these cells might did not affect experiment results for follow reason. It was reported that NKG2D was also expressed on most NKT cells, macrophage cells, and subpopulations of *γδ*T cells, except NK cells. NKT cells, macrophage cells, and *γδ*T cells could also kill tumor cells like NK cells [23, 29–31], but the concentration of NKT and *γδ*T cells in PBMC is rare. Macrophage cells were adherent growth. We gained the NK cells solution from supernatant and liquor. Thus, these cells might not affect experimental results also. We also found that the concentration of NK cells (18%) was a little higher than normal range (5–10%) in PBMC, which might be the effect of rhIL-2 and PHA added into PBMC solution.

There are three types of VIP/PACAP receptors, VPAC1, VPAC2, and PAC1. The different receptors bond VIP with different affinities. VPAC1 showed the most remarkable affinities to VIP among all the receptors [11, 21]. So, VPAC1 might play a major role. In this study, we observed the VPAC1 in NK and MKN45 cells. However, if any other VIP receptors took role in this signal pathway, they would need further study.

From above, we concluded that the VIP could inhibit NK cells' cytotoxicity on MKN45, which might get through downregulating the expressions of NKG2D, DAP10, and NF-*κ*B in NK cells. So, gastric cancer cells might escape organism immune cleaning by secreting VIP to inhibit NKG2D signal pathway of NK cells. However, are there any other members except NKG2D, DAP10, and NF-*κ*B in this signal pathway? How did NK cells affect gastric adenocarcinoma *in vivo*? Those questions need further study. Our finding provide a new insight into the importance of VIP in gastric cancer progression. The VIP antagonist may be helpful antigastric cancer drug by targeted VIP.

## Figures and Tables

**Table 1:** List of primers.

Beta-actin primer 1 (sense)	5′CGCTACAGCTTCACCACCAC3′
Beta-actin primer 2 (antisense)	5′TACTCCTGCTTGCTGATCCAC3′
VIP primer 1 (sense)	5′TCCTTGTGCTCCTGACTCTT3′
VIP primer 2 (antisense)	5′GACTGCATCTGAGTGACGTT3′
VPAC1 primer 1 (sense)	5′CTATGTGCAGATGATCGAGG3′
VPAC1 primer 2 (antisense)	5′GAAGAGGTGCATGTGGATGT3′
NKG2D primer 1 (sense)	5′TTCTGCTGCTTCATCGCTGT3′
NKG2D primer 2 (antisense)	5′GGTGAGAGAATGGAGCCATC3′
DAP10 primer 1 (sense)	5′CAGTCCACCATGATCCATCT3′
DAP10 primer 2 (antisense)	5′TGCCTGGCATGTTGATGTAG3′
NF-*κ*Bp65 primer 1 (sense)	5′GAGAGGAGCACAGATACCAC3′
NF-*κ*Bp65 primer 2 (antisense)	5′CACAGCATTCAGGTCGTAGT3′

**Table 2:** The comparison of NK cells purity between group A and B.

Group	*n *	Purification of NK cells (%)	
A	10	60.583	*X* ^2^ = 36.750
B	10	18.508	*P* < 0.01

Group A: NK cells purity after purified from PBMC by CDC method. Group B: NK cells purity in PBMC.

**Table 3:** The cytotoxicity of NK cells to MKN45 cells and VIP effect on it.

Group	*n *	OD_490_
A: MKN45 + NK + VIP (1 × 10^−5^)	12	0.161 ± 0.024^ab^
B: MKN45 + NK + VIP (1 × 10^−6^)	12	0.157 ± 0.017^ab^
C: MKN45 + NK + VIP (1 × 10^−7^)	12	0.138 ± 0.022^abc^
D: MKN45 + VIP (1 × 10^−5^)	12	0.196 ± 0.019^b^
E: MKN45 + VIP (1 × 10^−6^)	12	0.200 ± 0.028^b^
F: MKN45 + VIP (1 × 10^−7^)	12	0.201 ± 0.025^b^
G: MKN45 + NK	12	0.106 ± 0.016^a^
H: MKN45 (blank control)	12	0.209 ± 0.026

*F* = 31.533, *P* < 0.01. a stands for *P* < 0.05 compared with group H. b stands for *P* < 0.05 compared with group G. c stands for *P* < 0.05 compared with group A.

**Table 4:** The cytotoxicity of VIP in 10^−6^ mol/L and its antagonist in 10^−4^ to 10^−6^ mol/L concentration for 48 h on the growth of NK cells to MKN45 cells.

Group	*n *	OD_490_
A: MKN45 + NK + VIP + antagonist (1 × 10^−4^)	12	0.114 ± 0.017^acd^
B: MKN45 + NK + VIP + antagonist (1 × 10^−5^)	12	0.115 ± 0.018^acd^
C: MKN45 + NK + VIP + antagonist (1 × 10^−6^)	12	0.141 ± 0.025^abce^
D: MKN45 + antagonist (1 × 10^−4^)	12	0.195 ± 0.024^bde^
E: MKN45 + antagonist (1 × 10^−5^)	12	0.197 ± 0.023^bde^
F: MKN45 + antagonist (1 × 10^−6^)	12	0.194 ± 0.024^bde^
G: MKN45 + NK + VIP	12	0.156 ± 0.019^abc^
H: MKN45 + VIP	12	0.196 ± 0.027^b^
I: MKN45 + NK	12	0.104 ± 0.024^a^
J: MKN45 (blank control)	12	0.208 ± 0.027

*F* = 36.751, *P* < 0.01. a stands for *P* < 0.05 compared with group J. b stands for *P* < 0.05 compared with group I. c stands for *P* < 0.05 compared with group H. d stands for *P* < 0.05 compared with group G. e stands for *P* < 0.05 compared with group B.

**Table 5:** The cytotoxicity of VIP or antagonist in 1 × 10^−6^ mol/L, 1 × 10^−5^ mol/L concentration, respectively, on the growth of NK cells to MKN45 cells for 24 h, 48 h, or 72 h.

Time (h)	*n *	OD_490_					
		A	B	C	D	E	F
24	12	0.115 ± 0.320	0.102 ± 0.028	0.124 ± 0.031	0.121 ± 0.037	0.093 ± 0.029	0.126 ± 0.038
48	12	0.115 ± 0.021*	0.153 ± 0.017^∗△^	0.197 ± 0.015^△^	0.200 ± 0.028^△^	0.106 ± 0.016*	0.209 ± 0.026^△^
72	12	0.319 ± 0.100*	0.381 ± 0.109	0.375 ± 0.097	0.381 ± 0.120	0.346 ± 0.124*	0.426 ± 0.120^△^

*P < 0.05, compared with group F in same time. ^△^ *P* < 0.05, compared with group E in same time. Group A: MKN45 + NK + VIP + antagonist; group B: MKN45 + NK + VIP; group C: MKN45 + antagonist; group D: MKN45 + VIP; group E: MKN45 + NK; and group F: MKN45 (control).

**Table 6:** The expression of NKG2D, DAP10, and NF-*κ*B in NK cells incubated with drug.

Group	*n *	NKG2D protein expression				NKG2D mRNA	DAP10 protein expression				DAP10 mRNA	NF-*κ*B protein expression				NF-*κ*B p65 mRNA
		−	+	++	+++	expression (IOD)	−	+	++	+++	expression (IOD)	−	+	++	+++	expression (IOD)
A	10	0	7	3	0	0.232 ± 0.024	0	0	8	2	0.415 ± 0.009	1	7	2	0	0.201 ± 0.014
B	10	0	1	8	1	0.554 ± 0.031	0	0	3	7	0.617 ± 0.013	0	0	8	2	0.495 ± 0.015
C	10	0	0	4	6	0.742 ± 0.080	0	0	2	8	0.625 ± 0.022	0	0	5	5	0.504 ± 0.027

		*H* = 16.447				*F* = 227.967	*H* = 8.136				*F* = 514.408	*H* = 18.051				*F* = 680.369
		*P* = 0.000				*P* = 0.000	*P* = 0.017				*P* = 0.000	*P* = 0.000				*P* = 0.000

Group A: NK cells + VIP; group B: NK cells + VIP + VIP antagonist; and group C: NK cells (control). IOD: the integrated optical density ratio of the target genes (NKG2D, DAP10, NF-*κ*B) to *β*-Actin.

## References

[1] Caligiuri MA (2008). Human natural killer cells. *Blood*.

[2] Jamieson AM, Diefenbach A, McMahon CW, Xiong N, Carlyle JR, Raulet DH (2002). The role of the NKG2D immunoreceptor in immune cell activation and natural killing. *Immunity*.

[3] Guerra N, Tan YX, Joncker NT (2008). NKG2D-deficient mice are defective in tumor surveillance in models of spontaneous malignancy. *Immunity*.

[4] Billadeau DD, Upshaw JL, Schoon RA, Dick CJ, Leibson PJ (2003). NKG2D-DAP10 triggers human NK cell-mediated killing via a Syk-independent regulatory pathway. *Nature Immunology*.

[5] Garrity D, Call ME, Feng J, Wucherpfennig KW (2005). The activating NKG2D receptor assembles in the membrane with two signaling dimers into a hexameric structure. *Proceedings of the National Academy of Sciences of the United States of America*.

[6] Fukumoto Y, Ikeguchi M, Matsumoto S (2006). Detection of cancer cells and gene expression of cytokines in the peritoneal cavity in patients with gastric cancer. *Gastric Cancer*.

[7] Said SI, Mutt V (1970). Polypeptide with broad biological activity: isolation from small intestine. *Science*.

[8] Delgado M, Ganea D (2013). Vasoactive intestinal peptide: a neuropeptide with pleiotropic immune functions. *Amino Acids*.

[9] Valdehita A, Carmena MJ, Bajo AM, Prieto JC (2012). RNA interference-directed silencing of VPAC1 receptor inhibits VIP effects on both EGFR and HER2 transactivation and VEGF secretion in human breast cancer cells. *Molecular and Cellular Endocrinology*.

[10] Li GH, Qian W, Song GQ, Hou XH (2007). Effect of vasoactive intestinal peptide on gastric adenocarcinoma. *Journal of Gastroenterology and Hepatology*.

[11] Langer I, Robberecht P (2007). Molecular mechanisms involved in vasoactive intestinal peptide receptor activation and regulation: current knowledge, similarities to and differences from the A family of G-protein-coupled receptors. *Biochemical Society Transactions*.

[12] Schulz S, Röcken C, Mawrin C, Weise W, Höllt V, Schulz S (2004). Immunocytochemical identification of VPAC1, VPAC2, and PAC1 receptors in normal and neoplastic human tissues with subtype-specific antibodies. *Clinical Cancer Research*.

[13] Diebold SS (2009). Activation of dendritic cells by toll-like receptors and C-type lectins. *Handbook of Experimental Pharmacology*.

[14] Chorny A, Gonzalez-Rey E, Fernandez-Martin A, Pozo D, Ganea D, Delgado M (2005). Vasoactive intestinal peptide induces regulatory dendritic cells with therapeutic effects on autoimmune disorders. *Proceedings of the National Academy of Sciences of the United States of America*.

[15] Goetzl EJ, Chan RC, Yadav M (2008). Diverse mechanisms and consequences of immunoadoption of neuromediator systems. *Annals of the New York Academy of Sciences*.

[16] Lv B, Tang Y, Chen F, Xiao X (2009). Vasoactive intestinal peptide and pituary adenylate cyclase-activating polypeptide inhibit tissue factor expression in monocyte in vitro and in vivo. *Shock*.

[17] Szliter EA, Lighvani S, Barrett RP, Hazlett LD (2007). Vasoactive intestinal peptide balances pro- and anti-inflammatory cytokines in the *Pseudomonas aeruginosa*-infected cornea and protects against corneal perforation. *Journal of Immunology*.

[18] Hejna M, Hamilton G, Brodowicz T (2001). Serum levels of vasoactive intestinal peptide (VIP) in patients with adenocarcinomas of the gastrointestinal tract. *Anticancer Research*.

[19] Ciccone E, Pende D, Viale O (1990). Specific recognition of human CD3^−^CD16^+^ natural killer cells requires the expression of an autosomic recessive gene on target cells. *Journal of Experimental Medicine*.

[20] Tzaneva MA (2002). Endocrine cells in gastric carcinoma and adjacent mucosa. An immunohistochemical and ultrastructural study. *Histochemical Journal*.

[21] Yadav M, Goetzl EJ (2008). Vasoactive intestinal peptide-mediated Th17 differentiation: an expanding spectrum of vasoactive intestinal peptide effects in immunity and autoimmunity. *Annals of the New York Academy of Sciences*.

[22] Champsaur M, Lanier LL (2010). Effect of NKG2D ligand expression on host immune responses. *Immunological Reviews*.

[23] Raulet DH (2003). Roles of the NKG2D immunoreceptor and its ligands. *Nature Reviews Immunology*.

[24] Zafirova B, Wensveen FM, Gulin M, Polić B (2011). Regulation of immune cell function and differentiation by the NKG2D receptor. *Cellular and Molecular Life Sciences*.

[25] Tato CM, Mason N, Artis D (2006). Opposing roles of NF-*κ*B family members in the regulation of NK cell proliferation and production of IFN-*γ*. *International Immunology*.

[26] Wan F, Lenardo MJ (2010). The nuclear signaling of NF-*κ*B: current knowledge, new insights, and future perspectives. *Cell Research*.

[27] Nakashima Y, Deie M, Yanada S, Sharman P, Ochi M (2005). Magnetically labeled human natural killer cells, accumulated in vitro by an external magnetic force, are effective against HOS osteosarcoma cells. *International Journal of Oncology*.

[28] Piroozmand A, Hassan ZM (2010). Evaluation of natural killer cell activity in pre and post treated breast cancer patients. *Journal of Cancer Research and Therapeutics*.

[29] Yoshida Y, Nakajima J, Wada H, Kakimi K (2011). *γδ* T-cell immunotherapy for lung cancer. *Surgery Today*.

[30] Maasho K, Opoku-Anane J, Marusina AI, Coligan JE, Borrego F (2005). Cutting edge: NKG2D is a costimulatory receptor for human naive CD8^+^ T cells. *Journal of Immunology*.

[31] Girardi M, Oppenheim DE, Steele CR (2001). Regulation of cutaneous malignancy by *γδ* T cells. *Science*.

